# The accuracy–utility gap: Why AI accuracy does not guarantee clinical benefit in digital pathology

**DOI:** 10.1016/j.jpi.2026.100751

**Published:** 2026-10-09

**Authors:** Romina Battiato, Felipe Montoya Silva, Mylene Cabrera Morales, Deyana Salazar Romero, Martín H. Benites

**Affiliations:** a Servicio de Anatomía Patológica, Hospital Regional Dr. Franco Ravera Zunino, Av. Lib-ertador Bernardo O'Higgins 3065, Rancagua, Región del Libertador General Bernardo O'Higgins, Chile; b Centro de Pacientes Críticos, Clínica Las Condes, Santiago, Chile; c Escuela de Medicina, Facultad de Medicina, Universidad Finis Terrae, Santiago, Chile

**Keywords:** Artificial intelligence, Digital pathology, Computational pathology, Clinical utility, Clinical translation, External validation, Workflow integration

## Abstract

Artificial intelligence (AI) in digital pathology has repeatedly achieved expert-level diagnostic accuracy under controlled validation conditions. However, its clinical translation—reflected in regulatory approval, routine adoption, changes in diagnostic decisions, or demonstrated patient benefit—remains inconsistent. This viewpoint argues that this discrepancy arises because diagnostic accuracy is a property of a model under specified conditions, whereas clinical utility emerges from the entire sociotechnical pathway in which the model is deployed. Building on recent work on standardization, system selection, institutional validation, and trial governance, we integrate these complementary layers into a continuous tissue-to-outcome pathway comprising six stages: tissue, image, model, workflow, decision, and outcome, with each stage explicitly linked to the claim it can support. Variability introduced at any stage may remain undetected and propagate downstream, potentially affecting clinical decisions and patient care. Accordingly, evidence generated at one stage cannot substitute for that required to support claims at subsequent stages. We therefore propose a stage-linked evidence map that identifies the principal risk and evidence required at each stage, while making explicit which stronger downstream claims remain unsupported. This framework enables reviewers and institutions considering adoption to identify where a study has addressed the accuracy–utility gap and where uncertainty remains. Evaluating AI across the complete pathway reframes the evidentiary requirements for clinical translation: high diagnostic accuracy is necessary, but insufficient, to establish meaningful clinical utility.

## Introduction

Artificial intelligence (AI) in digital pathology has repeatedly achieved expert-level diagnostic performance under controlled validation conditions, including tumor detection, grading, and quantitative biomarker assessment.^1^ Selected prostate cancer detection systems have undergone large multi-institutional clinical validation and, on that basis, received the first FDA marketing authorization for an AI-based digital pathology diagnostic aid.^2^ Yet, outside these narrow, well-resourced deployments, successful clinical translation, whether measured as regulatory approval, routine adoption, a change in diagnostic decisions, or demonstrated patient benefit, remains inconsistent.

High area-under-the-curve values, reported in isolation, do not establish whether an algorithm changes the diagnosis, treatment decisions, workflow, or patient outcomes, and systematic reviews of the field continue to report substantial gaps between internal validation metrics and any measure of downstream clinical impact.^3^ Accuracy is a property of a model under specified conditions, whereas utility is a property of the entire sociotechnical pathway in which the model operates.^4^ This raises the question of not whether AI can be accurate, but why accuracy so often fails to become value at the point of care.

Recent works have addressed complementary aspects of this problem. Chauhan et al. mapped the standard ecosystem—including DICOM, ICC, and IHEPaLM—required for interoperable and sustainable AI deployment.^5^ Montezuma et al. provided a structured guide for the pre-deployment evaluation of digital pathology systems, formalizing the criteria used to select infrastructure that will shape the image acquisition and workflow environment in which AI models are deployed.^6^ Janowczyk et al. operationalized, through a fully worked single-center example, the validation, risk assessment, accreditation, deployment, and post-deployment monitoring of a clinical digital pathology tool.^7^ Sebastian et al. mapped the regulatory landscape governing digital pathology and AI within clinical trials, linking compliance requirements to patient risk and intended use.^8^ Each of these works addresses an essential segment of the translational pathway—standardization, procurement, institutional validation, and trial governance—and together, they define many of the technical, organizational, and regulatory conditions that make AI deployment possible. Building on these contributions, this viewpoint connects these complementary layers within a continuous tissue-to-outcome pathway and links each transition to the clinical evidence required to justify progressively stronger claims of translation across both regulated research and routine clinical deployment. This stage-linked framework is summarized in Fig. 1.

**Fig. 1:**
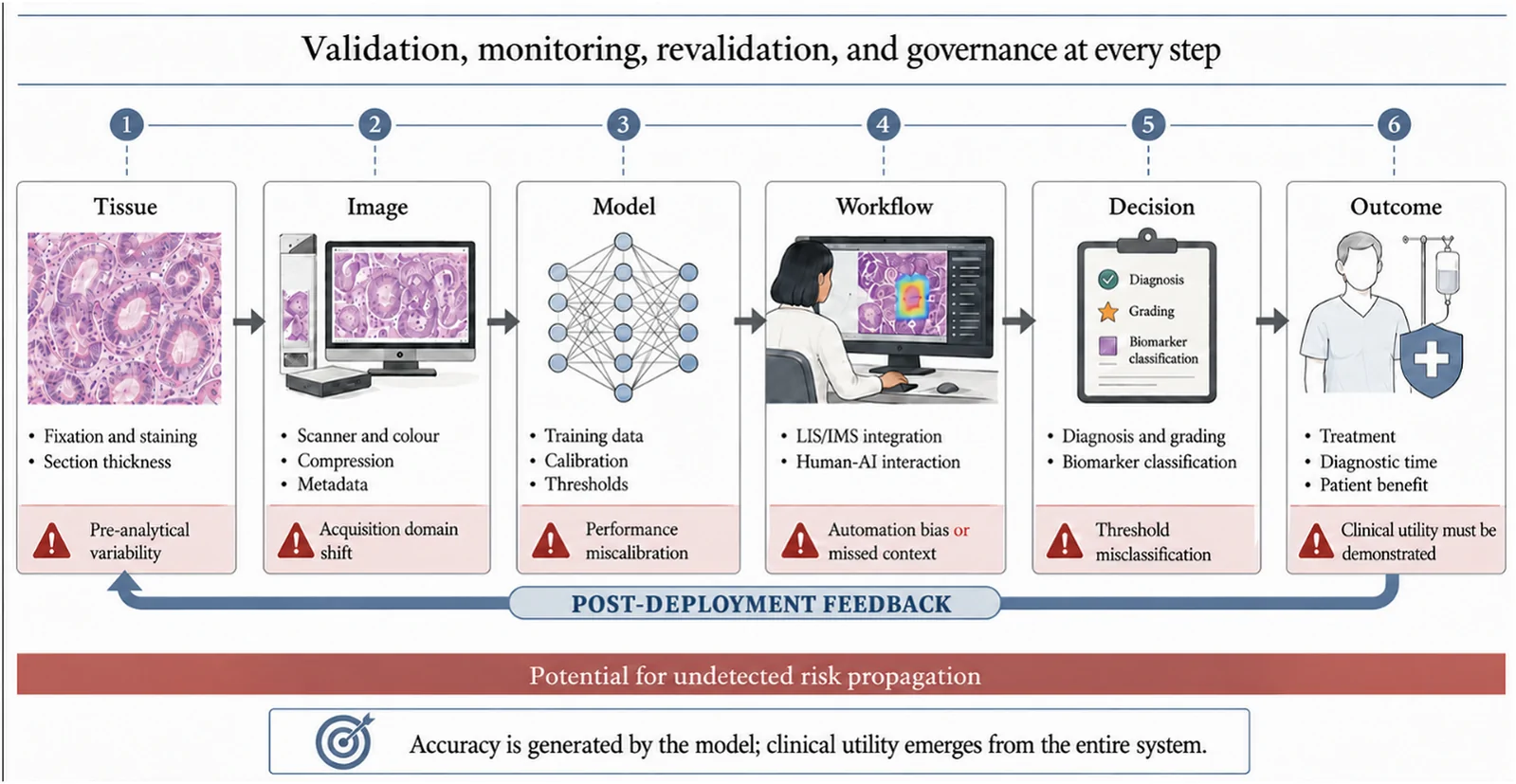
Potential for risk propagation and post-deployment feedback across the digital pathology lifecycle. The tissue-to-outcome pathway comprises six stages—tissue, image, model, workflow, decision, and outcome—each of which can introduce variability capable of propagating downstream and influencing clinical decisions or patient care. Such propagation is neither inevitable nor necessarily cumulative: quality control, external or local validation, calibration, human review, and governance may detect and interrupt failures at different stages. The blue return arrow represents feedback from post-deployment evidence and monitoring to relevant upstream stages. Because preanalytical processes, acquisition systems, software or model versions, patient populations, and clinical workflows may change over time, post-deployment monitoring may identify performance drift or changes that call previously supported claims into question, prompting, where indicated, recalibration, revalidation, modification, or restriction of use. Monitoring and revalidation therefore operate across the entire pathway as part of a continuing lifecycle rather than as activities confined to the outcome stage. AI, artificial intelligence; IMS, image management system; LIS, lab information system. (For interpretation of the references to color in this figure legend, the reader is referred to the web version of this article.)

## From tissue to outcome: Where variability can propagate

Every stage of the digital pathology pathway can introduce variability that is inherited by the next stage and, if left undetected, may ultimately influence clinical decisions. Fixation time, staining protocols, section thickness, and tissue preparation modify morphology before an image is acquired. Scanner optics, color calibration, and image compression then transform that already-variable tissue into a digital representation whose appearance depends partly on acquisition hardware rather than biology alone.^9^, ^10^ As a result, models operating outside the acquisition conditions under which they were developed and validated may generate high-confidence predictions despite encountering previously unrepresented sources of variation.

Importantly, this propagation is neither inevitable nor irreversible. Evidence from the PANDA challenge, an independent population-based validation study, and the real-world implementation of a clinical-grade prostate cancer detection system indicates that transportability across multinational and multi-institutional cohorts is achievable when supported by appropriately designed external validation studies.^11^, ^12^, ^13^ External validation does not eliminate domain shift; rather, it can reveal such shifts before deployment, enabling recalibration, model adaptation, or restriction of use. Conversely, in the absence of external validation, site-specific acquisition characteristics and digital histology signatures may remain undetected, resulting in clinically relevant performance degradation after deployment.^14^

The same principle extends beyond conventional supervised models. Foundation models pretrained on large repositories of whole-slide images have demonstrated strong performance across multiple downstream tasks, including molecular prediction and outcome inference. However, recent independent benchmarking suggests that downstream performance depends less on pre-training scale than on the representativeness of the underlying datasets and their alignment with the intended deployment environment.^15^ Consequently, even highly capable foundation models do not eliminate the need for local validation, recalibration, and continuous performance monitoring across different scanners, labs, and patient populations.

The pathway is illustrated primarily through image-based predictive AI, but its underlying logic is not restricted to such models and extends to pathology decision-support systems incorporating structured, textual, molecular, multimodal, or pathologist-generated inputs. For systems whose inputs extend beyond tissue-derived images, the upstream stages should be interpreted according to how each data source is generated, represented, and quality-controlled. Accordingly, the evidence and performance measures required at each stage should reflect the type and intended use of the AI system rather than a single set of metrics. Discrimination, calibration, and prespecified decision thresholds are relevant to predictive classifiers, whereas generative, retrieval-based, or workflow-support systems may require different measures, including factual reliability, task-specific error rates, failure detection, human–AI interaction, and downstream effects on clinical decisions.^16^, ^17^ The framework therefore specifies the evidentiary function of each stage rather than prescribing uniform performance metrics across AI systems.

Therefore, the central issue is not whether variability exists but where it is detected and interrupted before it influences patient care. The clinical utility of AI-based systems depends on detecting and controlling relevant sources of variability throughout the sociotechnical pathway rather than on algorithmic accuracy alone.

## How the stage-linked evidence map complements existing frameworks

Several recent frameworks address complementary aspects of clinical translation. The Digital Pathology Association's 2026 recommendation statement provides pathology-specific guidance on AI validation, implementation, performance verification, and assessment of clinical utility within clinical labs.^18^ FUTURE-AI offers a broader, cross-domain, lifecycle-oriented consensus framework organized around six guiding principles: fairness, universality, traceability, usability, robustness, and explainability.^16^ In computational pathology, Cai et al. described the persistent mismatch between algorithmic capability and routine clinical integration as an “adoption paradox” and proposed a three-stage maturity model spanning algorithmic capability, system integration, and institutional adoption.^17^

These frameworks share a central premise with the present viewpoint: algorithmic accuracy is necessary but does not establish successful deployment or clinical benefit. Our framework does not introduce this accuracy–utility gap as a new concept, nor does it replace existing guidance on validation, trustworthiness, implementation, or adoption. Instead, it organizes clinical translation as a sequence of evidence-dependent questions. Does the system perform accurately under defined conditions? Does that performance persist across populations and settings? Can the tool be safely integrated into clinical workflow? Once integrated, does it actually change clinical decisions? And, ultimately, do those changes improve patient outcomes? Each question requires different evidence, and answering one does not answer the next. Thus, each step represents a distinct evidentiary transition: demonstrating performance does not establish generalizability, demonstrating generalizability does not establish safe clinical integration, and demonstrating integration does not establish an effect on clinical decisions or patient outcomes.

The stage-linked evidence map operationalizes these transitions across the six-stage tissue-to-outcome pathway by identifying, at each stage, what has been demonstrated, the principal remaining risk and evidence required, and the stronger downstream claim that cannot yet be made (Table 1). Its purpose therefore differs from, but complements, existing frameworks: the DPA operationalizes lab validation and implementation, FUTURE-AI provides lifecycle principles, and the adoption paradox describes barriers to clinical integration. The present map instead makes explicit which claims the available evidence can support at each stage—and which stronger downstream claims still require demonstration.

**Table 1:** Stage-linked evidence requirements for progressively stronger claims of clinical translation.

Stage	Claim supported	Principal risk and evidence required	Claim not yet supported
Tissue	The tissue input preserves the biological signal required for the intended use.	*Principal risk:* Preanalytical variability. *Evidence required:* Documented fixation, processing, and staining provenance, with appropriate quality control and prespecified acceptance criteria.	Image transportability or model validity.
Image	The digital representation is transportable across the intended acquisition systems.	*Principal risk:* Acquisition domain shift. *Evidence required:* Validation across the intended scanners, institutions, file formats, compression settings, and acquisition conditions.	Model performance or calibration.
Model	Discrimination and calibration generalize to the intended population and use setting.	*Principal risk:* Dataset shift, model miscalibration, or inappropriate decision thresholds. *Evidence required:* External validation using representative data, calibration assessment, subgroup analysis, and prespecified threshold evaluation.	Safe workflow integration or clinical benefit.
Workflow	The system can be integrated and used safely and effectively in routine practice.	*Principal risk:* Poor integration, ineffective alerting, automation bias, or missed clinical context. *Evidence required:* Prospective evaluation under real-world conditions, including usability, human factors, workload, and error monitoring.	Appropriate changes in clinical decisions or improved outcomes.
Decision	AI assistance changes clinical judgment appropriately.	*Principal risk:* Miscalibrated trust, overreliance on AI output, or inappropriate override of correct human judgment. *Evidence required:* Human–AI interaction studies comparing decisions made with and without AI assistance and evaluating responses to both correct and erroneous advice.	Patient benefit.
Outcome	Deployment produces measurable net clinical benefit.	*Principal risk:* Reliance on surrogate endpoints, unmeasured harms, or failure to assess net benefit. *Evidence required:* Prospective comparative evaluation of effectiveness, safety, unintended consequences, and patient-centered outcomes.	Benefit across different populations, institutions, or workflows.

*Note:* For each stage in the tissue-to-outcome pathway shown in Fig. 1, the table identifies the claim that can be supported, the principal risk and corresponding evidence requirement, and the stronger claim that remains unsupported. Foundational prerequisites (infrastructure and governance) make safe deployment possible, whereas clinical-value determinants (representativeness, calibration, workflow integration, and outcome evidence) determine whether deployment produces clinical benefit. Evidence generated at one stage does not substitute for the evidence required at subsequent stages.

*Abbreviation:* AI, artificial intelligence.

This distinction matters because technical readiness and clinical utility are not equivalent. Standards, interoperability, security, and governance make deployment possible but do not establish that a model will remain valid in the population or workflow in which it is used. Architectural approaches may improve scalability and interoperability, including modular, standards-aligned designs separating image data from orchestration logic, as proposed by Gershkovich and subsequently discussed by Pantanowitz and Parwani.^19^, ^20^ Yet, even a fully DICOM-compliant, accredited deployment may produce misleading results if the deployment population, calibration procedure, or workflow context differs from those represented during development and validation. Conformance establishes technical readiness; it does not establish clinical validity or benefit.

The converse is equally important. A technically accurate algorithm characterized by discrimination, sensitivity, specificity, or area under the curve does not establish what happens when its output enters a real clinical workflow and influences a human reader. This distinction is not unique to AI. In a prospective multi-institutional comparison of four PD-L1 immunohistochemistry assays, scoring concordance among trained pathologists varied by assay and threshold, showing that decision-level performance cannot be inferred from analytical characterization alone.^21^ Human–AI interaction adds another layer: among 28 trained pathology experts estimating tumor cell percentages, erroneous AI advice overturned previously correct assessments in approximately 7% of consultations.^22^ Here, the failure emerged not from algorithmic performance alone, but from the interaction between the algorithm and the user.

These examples illustrate why evidence must remain linked to the stage at which a claim is made. Representative data are needed to establish generalizability; technical and governance safeguards support safe deployment; human–AI studies determine whether integration alters decisions appropriately; and prospective clinical studies are required to establish patient benefit. Evidence at one stage cannot substitute for evidence at another. The same logic applies whether a tool is evaluated as an investigational device under trial governance or deployed as a diagnostic aid in routine practice; what differs is how the relevant evidence is generated and reviewed, including formal GCP and regulatory processes in the former and predominantly institutional validation in the latter.^8^ Thus, claims of external validity, safe deployment, workflow benefit, and patient benefit are distinct claims requiring distinct evidence (Table 1).

## What this means for how the field reports evidence

Because clinical utility depends on multiple stages of the tissue-to-outcome pathway, its evaluation should not be limited to a single aggregate accuracy metric but should consider the evidence required at each relevant stage. Existing reporting frameworks, including STARD-AI for diagnostic accuracy studies and DECIDE-AI for early-stage clinical evaluation, already address many of these elements.^23^, ^24^ However, although they cover complementary components of clinical translation, they are not specifically designed to show what evidence supports each stage of the pathway, which risks remain unresolved, and which downstream claims cannot yet be made.

Studies making claims of clinical utility, transportability, or deployment readiness should therefore report the evidence relevant to each stage underlying those claims. This includes the provenance of source data and, where applicable, the acquisition conditions of training and evaluation data; the demographic and disease-prevalence representativeness of the evaluation cohort relative to the intended deployment population, because misdiagnosis risk has been shown to vary systematically across demographic subgroups in computational pathology models^25^; whether validation was performed using data external to the development institution; and task-appropriate performance measures reflecting the type and intended use of the AI system, including, for predictive classifiers, calibration at clinically relevant decision thresholds rather than discrimination alone. Claims extending beyond model performance require corresponding downstream evidence: deployment readiness should include evidence of safe workflow integration, claims of decision benefit should assess whether AI assistance changes clinical judgment appropriately, and claims of clinical utility should be supported by prospectively defined patient-level outcomes, as appropriate to the intended use.

Importantly, this pathway should be viewed as a continuing lifecycle rather than as a process that terminates at the outcome stage. Post-deployment monitoring is therefore a cross-cutting requirement. Changes in preanalytical processes, acquisition systems, software or model versions, patient populations, or workflows may invalidate previously supported claims and warrant surveillance for performance drift and, where indicated, revalidation. Evidence generated after deployment should feedback to the relevant upstream stages, allowing previously established claims to be reassessed as the system and its clinical environment evolve.^16^, ^17^, ^18^

A reviewer or adopting institution should therefore be able to determine which stages of the accuracy–utility pathway a study has evaluated, which claims are supported by the available evidence, and which remain uncertain. This principle applies both within and outside regulated trials: risk-based frameworks may define an intended use or permissible regulatory claims,^8^ but authorization alone does not establish that the evidence required for downstream clinical benefit has been generated.

This framework is conceptual rather than a systematic review or an empirically validated causal model. The transitions between stages are not necessarily linear, failures are not inevitably cumulative, and the evidence required at each stage will depend on the intended task, population, and clinical context. Prospective evaluation is therefore needed to determine whether applying the framework improves evidence appraisal, implementation decisions, or post-deployment surveillance across different AI systems and deployment settings.

## Conclusion

The future of computational pathology will depend not only on building increasingly accurate algorithms but also on demonstrating that accuracy survives the journey from tissue to treatment. The stage-linked evidence map proposed here complements existing validation and implementation frameworks by making explicit which claims the available evidence supports at each stage and where uncertainty remains. The critical question is no longer whether an AI system can perform its intended task accurately, but where along the clinical pathway technically accurate systems lose their capacity to improve patient outcomes and what evidence is required to show that those vulnerabilities have been addressed. Diagnostic accuracy remains a property of the model, whereas clinical utility emerges from the ecosystem in which the model is developed, validated, and deployed. Until performance across this ecosystem has been adequately demonstrated, high diagnostic accuracy will remain necessary, but never sufficient, for meaningful clinical impact.

## Funding

This research did not receive any specific grant from funding agencies in the public, commercial, or not-for-profit sectors.

## Declaration of generative AI and AI-assisted technologies in the manuscript preparation process

During the preparation of this work, the authors used Claude Fable 5 (Anthropic) to assist with language editing, improve the clarity and organization of the manuscript, and support the preparation of the explanatory diagram presented in Fig. 1. After using this tool/service, the authors reviewed and edited the content as needed and took full responsibility for the content of the published article.

## Declaration of competing interest

The authors declare that they have no known competing financial interests or personal relationships that could have appeared to influence the work reported in this article.
